# Reduced-order modeling for stochastic large-scale and time-dependent flow problems using deep spatial and temporal convolutional autoencoders

**DOI:** 10.1186/s40323-023-00244-0

**Published:** 2023-05-19

**Authors:** Azzedine Abdedou, Azzeddine Soulaimani

**Affiliations:** Department of Mechanical Engineering, Ecole de technologie superieure, 1100 Notre-Dame W., Montreal, H3C 1K3 QC Canada

**Keywords:** Uncertainty propagation, Reduced-order modeling, Deep learning, Convolutional autoencoders

## Abstract

A non-intrusive reduced-order model based on convolutional autoencoders is proposed as a data-driven tool to build an efficient nonlinear reduced-order model for stochastic spatiotemporal large-scale flow problems. The objective is to perform accurate and rapid uncertainty analyses of the flow outputs of interest for which the input parameters are deemed uncertain. The data are constituted from a set of high-fidelity snapshots, collected using an inhouse high-fidelity flow solver, which correspond to a sample of the uncertain input parameters. The method uses a 1D-convolutional autoencoder to reduce the spatial dimension of the unstructured meshes used by the flow solver. Another convolutional autoencoder is used for the time compression. The encoded latent vectors, generated from the two compression levels, are then mapped to the input parameters using a regression-based multilayer perceptron. The proposed model allows for rapid predictions for unseen parameter values, allowing the output statistical moments to be computed efficiently. The accuracy of the proposed approach is compared to that of the linear reduced-order technique based on an artificial neural network through two benchmark tests (the one-dimensional Burgers and Stoker’s solutions) and a hypothetical dam break flow problem, with an unstructured mesh and over a complex bathymetry river. The numerical results show that the proposed methods present strong predictive capabilities to accurately approximate the statistical moments of the outputs. In particular, the predicted statistical moments are oscillations-free, unlike those obtained with the traditional proper orthogonal decomposition method. The proposed reduction framework is simple to implement and can be applied to other parametric and time-dependent problems governed by partial differential equations, which are commonly encountered in many engineering and science problems.

## Introduction

Computational science has made great gains in efficiency due to the increases in high-performance computational resources and the emergence of novel methods, with significant achievements in scientific and industrial areas. Most of the computational mechanics problems are described by time-dependent and parameterized nonlinear partial differential equations. Their discretization over fine spatial meshes and for a high number of time steps leads to the so-called high-fidelity solutions, whose evaluation renders the computational techniques prohibitively expensive when dealing with multi-resolutions, as in optimization and uncertainty quantification [1, 2]. Reduced-order modeling (ROM) [3] has gained interest as a powerful methodology to reduce the cumbersome computational effort involved in high-fidelity solutions, while ensuring acceptable accuracy, particularly for situations that require real-time predictions.

One of the approaches most commonly adopted in reduced-order modeling is proper orthogonal decomposition (POD) [4, 5], which allows an approximation of the outputs using a linear combination of a limited number of basis functions [6, 7]. Many non-intrusive techniques have been proposed to compute the coefficients of a POD linear approximation using the data-driven concept without any modification of the governing equations [8–10]. Some of these methods are based on the stochastic framework and account for the variability stemming from the parametric domain by expressing the modal coefficients as a function of the stochastic basis functions, as in the polynomial chaos expansion method (POD-PCE) [11, 12] and the B-splines Bézier-element method (POD-BSBEM) [13]. Recent studies have explored the combination of the POD basis function with artificial neural networks (ANN) to construct an efficient regression framework for linear reduced-order modeling for time-dependent problems by learning the mapping between the time-parameter inputs and the modal coefficients of the POD basis function [14–16]. Despite the wide adoption of linear reduced-order modeling in approximating the outputs for parametric and time-dependent problems, they still encounter difficulties in accurately capturing the dynamics of some complex physical problems (i.e., those with strong hyperbolic behavior or shock propagation waves) without considerably increasing the number of reduced basis functions and thus compromising the low dimensionality aspect [17, 18].

To overcome the limitations of the techniques based on linear dimensionality reduction [19], recent methods based on nonlinear manifolds have gained interest in the field of dimensionality reduction. Some of these approaches are based on the recently developed algorithms of deep learning technology that are effective in learning sophisticated abstract features [20–23]. The autoencoder-based method (AE), a special type of neural network (NN) with two parts, the encoder and the decoder, trained jointly, has been successfully introduced as a nonlinear dimensionality reduction framework with an effective nonlinear relationship between variables [24, 25]. Some limitations concerning classical autoencoders based on dense layers have been reported in the literature, due to the drastic increase in the number of trainable parameters when the dimension of the input dataset becomes large [19, 26]. Convolutional autoencoders (CAEs) have emerged as an alternative to AEs in nonlinear compression manifolds. Their structure comprises several operations such as convolution-deconvolution, pooling-upsampling, and a multilayer perceptron layer (MLP) [27, 28]. CAEs offer the possibility of sharing coefficients and local patch connections, allowing a significant reduction in the number of trainable parameters. It is also possible to impose conditions on the latent space as proposed in the $\mathcal {\beta }$-variational autoencoder-CNN architecture [29] where the latent vectors are ranked based on their contribution to the construction and learned to be nearly non-orthogonal.

To make full use of temporal information, many sequence networks, including the recurrent neural network (RNN) [30] and its variants (e.g. LSTMs, GRUs, TCN, Transformer etc.) have been widely used. The idea behind recurrent neural networks (RNNs) is to apply the weight sharing operations at each time instant by involving the current state (previous history) as well as the value of the current input. In this way, a network can scale well (and rapidly) to sequences of different lengths. The key idea behind the popular recurrent network, long-short term memory, (LSTM) [31] is the so-called cell state, which helps to overcome the problems associated with the vanishing/exploding phenomena that are caused by the long-term dependencies within the network. Some configurations combine CAEs with LSTMs or TCNs [19, 32–35] to provide a tool for time predictions over the latent space.

This paper proposes a non-intrusive reduced order modeling (NIROM) framework for parametrized and time-dependent flow problems. The main objective is to perform accurate and rapid uncertainty analyses of the outputs of fluid dynamic flows for which the input parameters are deemed uncertain. This data-driven approach relies on the attractive features of convolutional autoencoders (CAEs) to reduce the dimensionality of high-fidelity solutions collected from numerical solvers with large-scale meshes. The method, referred to as NIROM-CAEs, is based on two compression levels provided by 1D-autoencoders. The first 1D-encoder reduces the spatial dimension by encoding the input dataset along the spatial dimension to generate a latent space. A second 1D-encoder convolves the latent space along the temporal dimension to output the final spatiotemporal encoded latent vector, which is mapped to the input parameters values through a multilayer perceptron (MLP). Therefore, for a new set of unseen parameters, the online predictive stage allows a rapid and accurate reconstruction of the original spatiotemporal dynamics through the trained 1D decoders. The proposed time prediction method is simple to implement and does not suffer from the vanishing or the exploding phenomena caused by long-term dependencies. The framework is applied to a stochastic treatment of two benchmark cases with univariate meshes, and to a hypothetical dam break flow over a natural river with complex bathymetry and two-dimensional unstructured meshes, which may present a challenging task for convolution autoencoders. By arranging the data into a vector according to the nodes’ numbering, a simple 1D-convolutinal autoencoder reveals sufficiently accurate results for the space compression step. The proposed method is compared with the more traditional approaches based on proper orthogonal decomposition. For solutions with strong gradients, the present method does not suffer from oscillations in the predicted statistical moments, unlike the POD-based methods. Therefore, the proposed framework offers interesting features in non-linear reduction modeling for physical problems with a high degree of complexity in their dynamics.

The paper is organized as follows: “Methodology” presents the fundamental framework of the proposed NIROM-CAEs, with detailed steps of the offline and online stages. Numerical test cases for assessing the performance and accuracy of the proposed method are provided in “Results and discussion”, followed by a summary and concluding remarks in “Conclusion”.

## Methodology

This section describes the fundamental framework of the proposed non-intrusive reduced model based on 1D convolutional autoencoders for space and time dimensions. The method belongs to the data-driven approaches, where high-fidelity solutions to large-scale and time-dependent physical problems are obtained from numerical solvers and gathered for both time and parameter sequences to construct snapshot matrices.

### Convolutional autoencoders (CAEs)

**Fig. 1:**
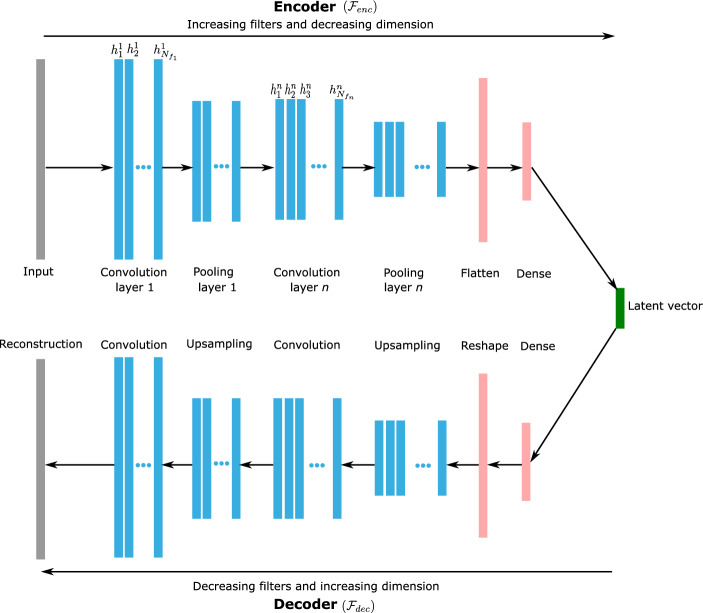
Schematic representation of 1D convolutional autoencoder architecture. The same structure of the 1D-CAE is adopted in the encoding and decoding processes for both the space and time dimensions

Convolutional autoencoders (CAEs) have gained interest as powerful nonlinear reduced-order modeling techniques with remarkable performances in the image recognition field. Convolutional layers have been introduced to overcome some of the limitations that classical autoencoders (AEs) based on dense layers may face when treating time-dependent problems with high dimensional inputs [19]. These layers are characterized by two properties: local connections and shared weights, thus allowing a feature map of the input and a limitation of the number of trainable parameters. Convolutional autoencoders have two distinct symmetrical parts: the first part, called the encoder ($\mathcal {F}_{enc}$), reduces the dimension of the input matrix by mapping with a latent space through a combination of successive convolutional, pooling, and fully connected layers; and the second part, the decoder ($\mathcal {F}_{dec}$), a combination of dense, upsampling, and convolutional layers, which maps the latent reduced-dimension vector to a larger-dimensional reconstruction of the input. A schematic representation of a 1D convolutional autoencoder architecture is presented in Fig. 1. It should be emphasized that the same structure of the 1D CAE is adopted in this study for the encoding and decoding processes for both space and time dimensions. Thus, the presentation of the framework of the proposed approach will concern mainly the 1D convolutional autoencoders.

**Fig. 2:**
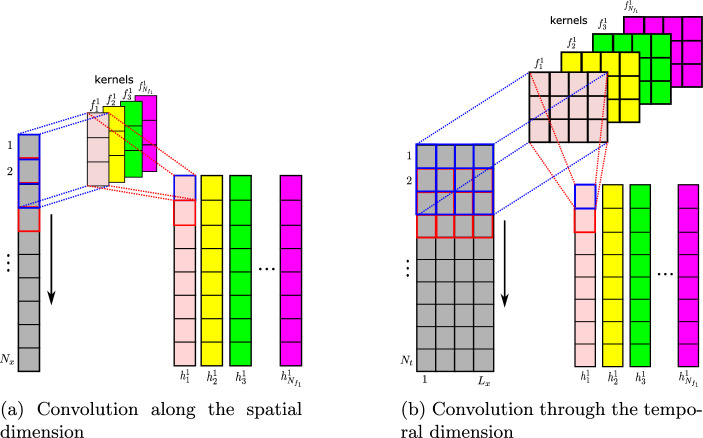
Schematic representation of the 1D-convolutional operation along the spatial and temporal dimensions. For interpretation of the references to color in this figure, the reader is referred to the web version of this article

A convolution layer represents a feature map where each unit in the layer is locally connected to a selected part of the previous layer via a kernel (filter) and an activation function. This operation allows the most dominant features from the input data to be extracted by applying a filter that moves with the vertical stride in the case of 1D convolution, as shown in Fig. 2. In this figure, a schematic representation of the 1D-convolution operation for a given layer *n* is detailed for both space and time directions. A mathematical formulation of a typical 1D-convolutional operation denoted by the symbol ($*$), shown in Figs. 1 and 2, can be expressed as follows [27, 36]:

1 $$\begin{aligned} h_{i}^{\ell }=\sigma \left( \textbf{H}^{\ell -1}*\textbf{f}_{i}^{\ell }+b_{i}^{\ell }\right) , \end{aligned}$$

where $h_{i}^{\ell } \in \mathbb {R}^{D_{\ell }\times 1}$ denotes the $i^{th}$ feature of the $\ell ^{th}$ layer, $\sigma$ is a nonlinear activation function, $\textbf{H}^{\ell -1}=\left[ h_{1}^{\ell -1},\,h_{2}^{\ell -1},\ldots ,h_{N_{f_{\ell -1}}}^{\ell -1}\right]$ stands for the convolution layer $\ell -1$, $\textbf{f}_{i}^{\ell }$ is the kernel of the layer $\ell$, and $b_{i}^{\ell }$ the bias parameter, with $i\in (1,\,N_{f_{\ell }})$ and $\ell \in (1,\,n)$. The number of feature column vectors in each layer $N_{f_{\ell }}$ corresponds to the number of kernels represented by different colors in Fig. 2, and the total number of layers *n* defines the depth of the convolution neural network (CNN). In addition to the convolutional operations, pooling layers (in this case, a max-pooling) are also integrated after each convolution layer to reduce the dimension of the convolved features by a fixed factor defined as a kernel size of the max-pooling layer, thus allowing the most dominant features of the local domain to be maintained [37]. The output features resulting from a max-pooling operation are illustrated in the encoder part of Fig. 1 for the $n^{th}$ layer, and denoted by $p_{i}^{n}$ with $i\in (1,\,N_{f_{n}})$.

### Non-intrusive reduced order model based convolutional autoencoders (NIROM-CAEs)

As mentioned above, the proposed approach combines convolutional autoencoders with the reduced order modeling concept and concerns the parametrized time-dependent problems with large-scale computational domains. The framework of the non-linear surrogate model is mainly based on a succession of two compression levels through 1D convolutions. The first level concerns the space dimension while the second deals with the compression of the time dimension of the latent space obtained from the former spatial compression.

#### Dataset construction

The model proposed in this work belongs to the data-driven approaches where the input dataset, known as a snapshot matrix, is constructed through a collection of high-fidelity solutions from the numerical solver $\left\{ \textbf{u}(\eta _{s},t_{j})\in \mathbb {R}^{N_{x}\times 1};\,j=1,\ldots ,N_{t};\,s=1,\ldots ,N_{s}\right\}$, where $\eta _{s}$ represents the $s^{th}$ value of the random parameter $\eta$ within its generated sample set with a size of $N_{s}$, following an appropriate probability density function $\varrho (\eta )$. $t_{j}$ denotes the $j^{th}$ time step within the temporal domain $t\in \mathcal {T}=\left[ 0,\;T \right]$ decomposed into $N_{t}$ time steps. The high fidelity solutions are collected from the numerical solver as a column vector $\textbf{u}\in \mathbb {R}^{N_{x}\times 1}$, where $N_{x}$ is the total number of the meshing nodes that cover the computational domain. The snapshot matrix, obtained by gathering these solution vectors, can be expressed as a 3D global matrix:

2 $$\begin{aligned} \varvec{\mathcal {U}}=\left[ \;\mathcal {U}_{1}\mid \ldots \mid \mathcal {U}_{s}\mid \ldots \mid \mathcal {U}_{N_{s}} \right] \in \mathbb {R}^{N_{s}\times N_{x}\times N_{t}}, \end{aligned}$$

where $\mathcal {U}_{s}\in \mathbb {R}^{N_{x}\times N_{t}}$ is a 2D solutions matrix corresponding to the parameter value $\eta _{s}$ over all the time steps, expressed as:

3 $$\begin{aligned} \mathcal {U}_{s}=\left[ \textbf{u}(\eta _{s},t_{1})\mid \ldots \mid \textbf{u}(\eta _{s},t_{N_{t}}) \right] \in \mathbb {R}^{N_{x}\times N_{t}}. \end{aligned}$$

It should be noted that the global snapshot matrix given by Eq. (2) is divided during the learning process into two sets: a training set ($80\,\%$) and a validation set ($20\,\%$) for the space and time autoencoders.

#### Spatial compression

The spatial compression represents the first compression level of the proposed technique; its goal is to reduce the dimension of the input dataset along the spatial dimension from $N_{x}$ to $L_{x}$ with $L_{x}\ll N_{x}$. Each snapshot matrix $\mathcal {U}_{s}\in \mathbb {R}^{N_{x}\times N_{t}}$, as given by Eq. (3), has its relation to the parameter value $\eta _{s}$ reshaped by considering its transpose $\mathcal {U}_{s}^{T}\in \mathbb {R}^{N_{t}\times N_{x}}$ on which the space encoder $\mathcal {F}_{x_{enc}}$ is applied along the spatial dimension as follows:

4 $$\begin{aligned} \mathcal {V}_{x_{s}}=\mathcal {F}_{x_{enc}}\left( \mathcal {U}_{s}^{T}\right) \in \mathbb {R}^{N_{t}\times L_{x}}, \end{aligned}$$

where $\mathcal {V}_{x_{s}}\in \mathbb {R}^{N_{t}\times L_{x}}$ represents the encoded latent space corresponding to the parameter value $\eta _{s}$, with $s=1,\,\ldots ,\,N_{s}$. It should be emphasized that for implementation purposes in the TensorFlow library [38], the dimension of the input dataset has been extended to a 3D tensor of a shape $(N_{t},\, N_{x},\,1)$ through the use of a reshape operator: $\mathcal {R}:\mathbb {R}^{N_{t}\times N_{x}}\mapsto \mathbb {R}^{N_{t}\times N_{x}\times 1}$ [26], where the third dimension stands for the number of channels, which is equal to one in this case. A schematic representation of the 1D convolution operation performed on the first convolution layer by the space encoder is depicted in Fig. 2a, where $N_{f_{1}}$ 1D-filters, whose width corresponds to the number of channels, convolve a local patch of the input dataset along the spatial dimension, represented by the vertical arrow, to generate the features of the convolved layer $h_{i}^{1},\,i=1,\,\ldots ,\,N_{f_{1}}$. As shown in Fig. 2a, each filter is distinguished by the same color as its corresponding column features generated when it convolves along the spatial direction. The detailed structures of the space autoencoders adopted for different test cases are reported in Tables 2 and 4.

#### Temporal compression

Once the spatial dimension is encoded, a second compression level is performed on the obtained latent space $\mathcal {V}_{x_{s}}\in \mathbb {R}^{N_{t}\times L_{x}}$ along the temporal dimension to reduce its size from $N_{t}$ to a given $L_{t}$ through a 1D time encoder $\mathcal {F}_{t_{enc}}$ that has the same structure as that shown in Fig. 1. The encoding process can be mathematically expressed as follows:

5 $$\begin{aligned} \mathcal {V}_{t_{s}}\in \mathbb {R}^{L_{t}}=\mathcal {F}_{t_{enc}}\left( \mathcal {V}_{x_{s}}\right) , \end{aligned}$$

where $\mathcal {V}_{t_{s}}\in \mathbb {R}^{L_{t}}$ is the encoded latent vector corresponding to the single parameter value $\eta _{s}$. The convolution operation of the first layer 1D-time encoder, expressed by Eq. (1), is presented in Fig. 2b, where $N_{f_{1}}$ filters convolve local patches of the input dataset $\mathcal {V}_{x_{s}}\in \mathbb {R}^{N_{t}\times L_{x}}$ along the temporal dimension ($N_{t}$). $L_{x}$ denotes the number of channels of the input dataset, which corresponds to the width of each filter. The detailed structures of the time autoencoders applied to the test cases addressed in this study are reported in Tables 3 and 5, along with information about the number of convolution layers, kernel sizes, and activation functions.

#### Regression based multilayer perceptron (MLP)

The third level of the proposed approach concerns the encoded latent vector $\mathcal {V}_{t_{s}}\in \mathbb {R}^{L_{t}}$, obtained through two compression levels of the input dataset $\mathcal {U}_{s}\in \mathbb {R}^{N_{x}\times N_{t}}$, which is mapped to the input parameter $\eta _{s}$ via a multilayer perceptron (MLP) regression. This regression, which links the input parameter to the final latent vector, composed of multiple stacked fully-connected layers, can be expressed as follows [32]:

6 $$\begin{aligned} \mathcal {V}_{t_{s}}=\Pi _{MLP}\left( \eta _{s}\right) . \end{aligned}$$

The detailed architecture of the MLP used in this work for the addressed test cases is presented in Table 6. The mathematical framework presented in subsections through represents the main steps of the offline stage of the proposed NIROM-CAEs technique.

It is worth mentioning that the proposed model was built and trained using the open-source deep learning library TensorFlow [38] using the Adam optimizer with its default parameters. To accelerate the convergence and optimize the training processes, the data used during the training process of the offline stage are all normalized with the same min-max scaling [32, 39]:

7 $$\begin{aligned} \tilde{u}^{s}=\frac{u^{s}-min(\textbf{u}_{j})}{max(\textbf{u}_{j})-min(\textbf{u}_{j})}-0.5\;;\quad j=1,\,\ldots ,\,N_{t};\quad s=1,\,\ldots ,\,N_{s}, \end{aligned}$$

with $\tilde{u}^{s}\in \left[ -0.5,\,0.5\right]$. The inverse transformation must also be applied to the predicted data matrix during the online stage to recover the original scaling.

#### Online surrogate prediction

Once the three models are trained during the offline stage described above, the online surrogate prediction becomes straightforward. A new set of the random parameters is generated following its probability distribution function $\varvec{\widehat{\eta }}=\lbrace \widehat{\eta }_{1},\,\ldots ,\,\widehat{\eta }_{N_{s'}}\rbrace$, and for each unseen value $\widehat{\eta }_{s}$, the encoded Spatiotemporal latent vector $\widehat{\mathcal {V}}_{t_{s}}$ is predicted through the MLP regression model: $\widehat{\mathcal {V}}_{t_{s}}\in \mathbb {R}^{L_{t}}=\Pi _{MLP}\left( \widehat{\eta }_{s}\right)$. This predicted latent space is then decoded through the time decoder $\mathcal {F}_{t_{dec}}$ to approximate the latent spatial space: $\widehat{\mathcal {V}}_{x_{s}}\in \mathbb {R}^{N_{t}\times L_{x}}=\mathcal {F}_{t_{dec}}\left( \widehat{\mathcal {V}}_{t_{s}}\right)$. Finally, the obtained latent space is decoded using the space decoder $\mathcal {F}_{x_{dec}}$ to generate the predicted input dataset: $\widehat{\mathcal {U}}_{s}\in \mathbb {R}^{N_{t}\times N_{x}\times 1}=\mathcal {F}_{x_{dec}}\left( \widehat{\mathcal {V}}_{x_{s}}\right)$, which is transformed to its final shape through the inverse reshape operator $\mathcal {R}^{-1}:\mathbb {R}^{N_{t}\times N_{x}\times 1}\mapsto \mathbb {R}^{N_{t}\times N_{x}}$. Thus, the predicted snapshot matrix corresponding to the single value parameter $\widehat{\eta }_{s}$ throughout the online stage can be expressed as follows:

8 $$\begin{aligned} \widehat{\mathcal {U}}_{s}=\mathcal {F}_{x_{dec}}\left( \mathcal {F}_{t_{dec}}\left( \Pi _{MLP}\left( \widehat{\eta }_{s}\right) \right) \right) . \end{aligned}$$

The statistical moments can be estimated via the constructed surrogate model of the stochastic output response $\widehat{u}$ as follows:

9 $$\begin{aligned} \mu _{\widehat{u}}=\mathbb {E}\left[ \widehat{u}\right] =\int _{\Xi }\widehat{u}\varrho (\widehat{\eta })d\widehat{\eta }, \end{aligned}$$

and

10 $$\begin{aligned} \sigma _{\widehat{u}}^{2}=\mathbb {E}\left[ \widehat{u}^{2}\right] - \mu _{\widehat{u}}^{2} =\left[ \int _{\Xi }\left( \widehat{u}\right) ^{2}\varrho (\widehat{\eta })d\widehat{\eta }\right] -\mu _{\widehat{u}}^{2} \end{aligned}$$

**Fig. 3:**
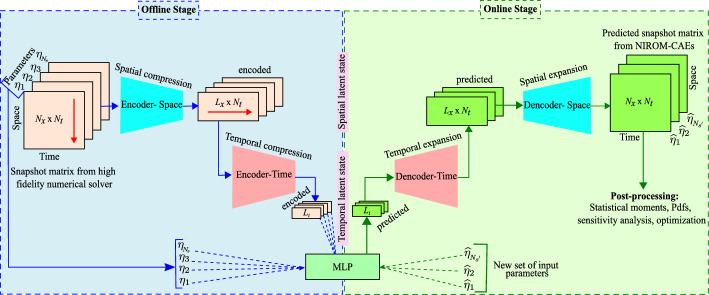
Flowchart illustrating the offline training and online predictive stages of the NIROM-CAEs model. The red arrow indicates the encoding direction (for interpretation of the references to color in this figure, the reader is referred to the web version of this article)

The fundamental framework of the proposed NIROM-CAEs approach is summarized in the flowchart presented in Fig. 3, which presents the most relevant steps of the offline training and online predictive stages.

The predictive abilities of the proposed NIROM-CAEs are assessed in comparison with the Latin Hypercube Sampling approach (LHS), whose results are considered as reference solutions. Another method based on a proper orthogonal decomposition concept combined with an artificial neural network (POD-ANN) is retained as a linear non-intrusive reduced order modeling technique for comparison purposes. A detailed presentation of the development and implementation steps of POD-ANN is beyond the scope of this paper, and the reader is encouraged to consult the corresponding references [14–16] for a deeper insight into the modeling procedure. It is worth mentioning that the architecture choice and hyperparameters selection were performed after a series of preliminary settings and a trial-and-error approach by combining, among others, the number of convolution layers, the number of filters, the kernel sizes, the activation functions, and the number of epochs. Indeed, several computations have been performed to select the appropriate 1D- spatial autoencoder structures for both spatial and temporal dimensions, and a tradeoff was made between the accuracy, the targeted compression levels, and the computational cost. A detailed description of the architectures and hyperparameters settings for each test case is presented in Tables 2, 3, 4 and 5.

## Results and discussion

This section presents the application of the proposed non-intrusive reduced order model to two well-known 1D benchmark test cases (Burgers’ and Stoker’s analytical problems) and to a hypothetical failure of a real dam case. The results are depicted to showcase the accuracy and efficiency of the proposed approach in approximating the statistical moments for time-dependent problems.

### One-dimensional Burgers equation test case

The first test case concerns the one-dimensional viscous Burgers’ equation for nonlinear convection-diffusion, expressed in its dimensionless form with the corresponding initial and Dirichlet boundary conditions as follows [40, 41]:

11 $$\begin{aligned} \frac{\partial {u}}{\partial {t}}+u\frac{\partial {u}}{\partial {x}}=\frac{1}{Re}\frac{\partial ^{2}{u}}{\partial {x^{2}}}\;,\qquad x\in \left[ 0,\;1\right] ,\qquad t\in \left[ 0,\;1\right] , \end{aligned}$$

with: $u(x,0)=\frac{x}{1+exp\left( \frac{Re}{16}(4x^{2}-1)\right) }$ and $u(0,t)=u(1,t)=0$. An analytical solution for the velocity field is given by [33]:

12 $$\begin{aligned} u(x,t)=\frac{\frac{x}{t+1}}{1+\sqrt{\frac{t+1}{t_{0}}}exp(Re\frac{x^{2}}{4t+4})}, \end{aligned}$$

with $t_{0}=exp(\frac{Re}{8})$. In this test case, the Reynolds number is considered as a random variable which follows a uniform distribution within its variability range $Re_{\mu =1000,\,\sigma =200}\in \mathcal {U}\left[ 654,\;1\,346\right]$. The sample used in this test case has a size $N_{s}=200$, randomly divided into two sets: one for training ($80\,\%$) and one for validation ($20\,\%$). For each selected value in the generated random parametric sets, the analytical solution given by Eq. (12) is evaluated over a discretized spatial domain with $N_{x}=1000$ nodes for all the $N_{t}=104$ time-steps to construct the training and validation high-fidelity snapshot matrices that are used to train the 1D spatial-CAE ($\mathcal {F}_{x}$) of the NIROM-CAEs approach, as described above.

The detailed architectures of the spatial encoder ($\mathcal {F}_{x_{enc}}$) and the spatial decoder ($\mathcal {F}_{x_{dec}}$), which consist of $1\,033\,773$ trainable parameters, are given in Table 2. The spatial encoder allows the compression of the spatial dimension from $N_{x}=1000$ nodes to a reduced-order latent space of dimension $L_{x}=50$ through a succession of 1D convolutional and max-pooling layers with associated nonlinear activation functions. The generated spatial latent space of dimension $N_{t}=104\times L_{x}=50$ serves as input to the 1D-time autoencoder ($\mathcal {F}_{t}$), which reduces the temporal dimension from $N_{t}=104$ to $L_{t}=10$. The detailed structures of both the encoder ($\mathcal {F}_{t_{enc}}$) and the decoder ($\mathcal {F}_{t_{dec}}$), with $177\,494$ trainable parameters, are shown in Table 3. The spatiotemporal latent space thereby obtained is then mapped with the input parameters using a multi-layer perceptron whose structure is given in Table 6 and which consists of $34\,570$ trainable parameters. The CAE-space, CAE-time, and the MLP were trained for 500, 1000 and 3000 epochs, respectively, and the decay of the training and validation losses during the training phase is depicted in Fig. 18.

**Fig. 4:**
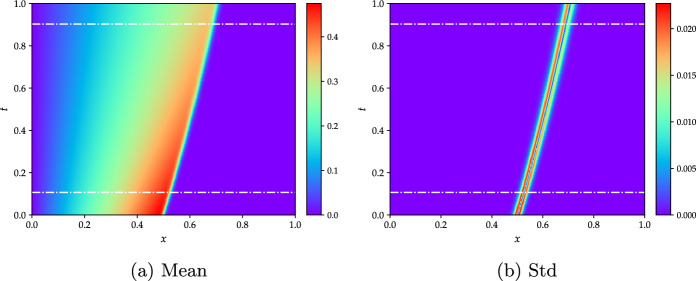
Contour plots of the mean and standard deviation of the Burgers’ analytical solution obtained from 5000 LHS realizations with uniform distribution $Re_{\mu =1000,\,\sigma =200}\in \mathcal {U}\left[ 654,\;1346\right]$. The two horizontal dashed white lines indicate the times for which the results in Fig. 5 are presented. (For interpretation of the references to color in this figure, the reader is referred to the web version of this article.)

The most relevant results for this test case obtained by the proposed NIROM-CAEs model are presented in terms of the statistical moments and relative $L^{2}$-error profiles, and compared with those from the POD-ANN and the LHS solutions. In Fig. 4, the 2D contours of the mean and standard deviation from the LHS method with 5000 realizations, considered as a reference solution, are presented in the space-time plane, where the discontinuity in the velocity field (Fig. 4a) and the variability surrounding its propagation with time (Fig. 4b) may present a challenging test case for the proposed reduced order modeling approach. The two horizontal dashed lines represent the time location for which the mean and the standard deviation profiles are displayed along with the spatial domain.

**Fig. 5:**
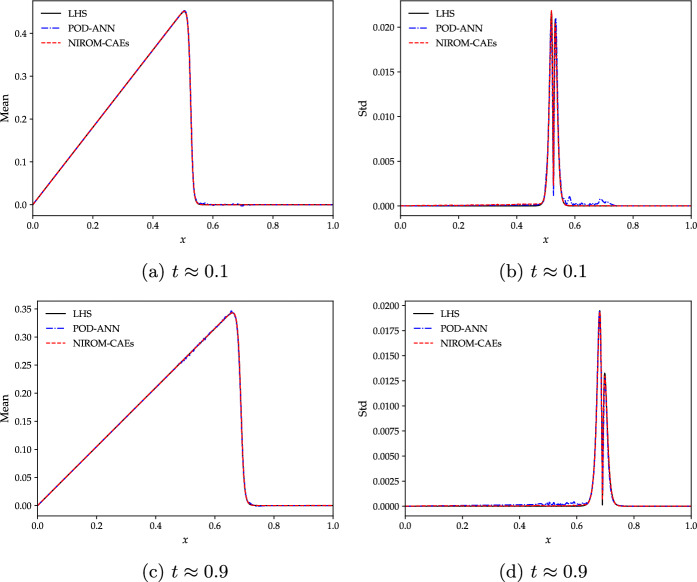
Comparison of the mean (left) and standard deviation (right) of the Burgers analytical solutions obtained with the POD-ANN and NIROM-CAEs approaches with those of the LHS reference solution (5000 realizations) at different times: $t\approx 0.1$ (**a**, **b**) and $t\approx 0.9$ (**c**, **d**)

Figure 5 reports the comparison of the mean and standard deviation profiles as a function of the *x*-coordinate at different time-steps ($t\approx 0.1$ and 0.9) obtained with NIROM-CAEs (with $L_{x}=50$ and $L_{t}=10$) and POD-ANN (with $\epsilon _{s}=\epsilon _{t}=10^{-10}$, which generates a latent space of dimension $L_{POD}=77$ modes), with those from the reference LHS solutions (with $N_{s}=5\,000$ realizations). These results show that the proposed non-intrusive surrogate model (NIROM-CAEs) predicts profiles of the mean (left column) and standard deviation (right column) that are in close agreement with those from the LHS reference solution. Conversely, an oscillatory behavior can be showcased in the statistical moments’ curves obtained with the POD-ANN technique with their pronounced deviations from the LHS solution curves. It should be emphasized that the predicted statistical moments from the NIRON-CAEs approach during the predictive online phase were obtained with new values of the random input parameter that differ from those used in the offline training phase. As evidenced by the results displayed in Fig. 5, the surrogate NIROM-CAEs technique allows a better approximation of the output quantities of interest due to the nonlinearities that effectively capture the dynamics of the viscous advection shock in contrast with the linear-based POD approach.

**Fig. 6:**
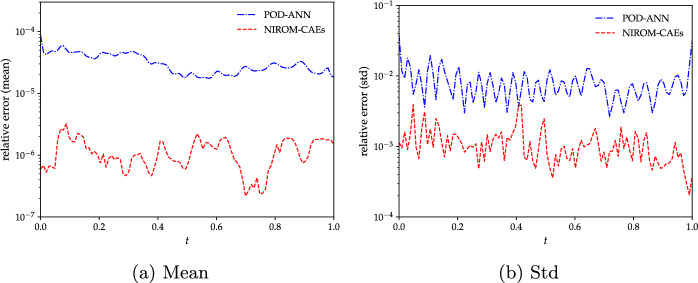
Variation of the $L^{2}$-relative error of the mean (left) and standard deviation (right) as a function of time obtained with the POD-ANN (blue) and NIROM-CAEs (red). Errors are evaluated in reference to the LHS reference solution obtained with 5000 realizations

To further investigate the performance of the proposed approach, the time trajectory of the spatial relative $L^{2}$-errors of the mean and standard deviation of the predicted solutions obtained by NIROM-CAEs are compared with those from the POD-ANN model. The relative errors are computed for the LHS reference solutions with $5\,000$ realizations over the whole set of computational nodes and for each time step. Its mathematical expression is defined as follows:

13 $$\begin{aligned} Err_{L^{2},\Phi }^{Surr}(t)= \sqrt{\frac{\sum _{i=1}^{N_{e}}\left( \Phi _{i,Surr}(t)-\Phi _{i,LHS}(t)\right) ^2}{\sum _{i=1}^{N_{e}}(\Phi _{i,LHS}(t))^2}}, \end{aligned}$$

where $\Phi$ denotes either the mean or the standard deviation, and *Surr* stands for NIROM-CAEs or POD-ANN. The comparison of the mean and standard deviation error profiles highlights the predictive abilities of the NIROM-CAEs model, which presents lower error values compared to those from the POD-ANN approach as shown in Fig. 6. These plots confirm the capacity of the proposed nonlinear reduced-order model to accurately estimate the statistics of the outputs, even for challenging time-dependent physical problems such as the advective viscous shock Burgers’equation.

### One dimensional dam break test case

**Fig. 7:**
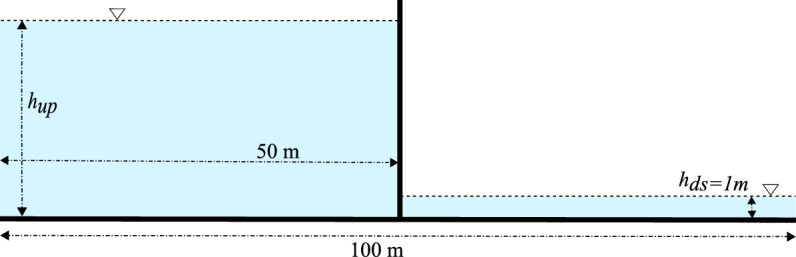
Schematic representation of the initial condition of the Stoker’s analytical solution of a 1D dam break over a wet flat bottom

The second test case concerns Stoker’s analytical solution [42], which describes the propagation and rarefaction wave resulting from a one-dimensional dam break over a wet flat frictionless bottom. The dynamic state is initiated by unequal water levels of both the upstream and downstream sides located in the middle of the studied domain of $100\;m$, as shown in Fig. 7. The mathematical expressions for the water level and the velocity of the parametrized Stoker’s analytical solution fields are given as follows [43, 44]:

14 $$\begin{aligned} h(x,t) = {\left\{ \begin{array}{ll} h_{up} \\ \frac{4}{9g}(\sqrt{g\,h_{up}}-\frac{x}{2t})^{2} \\ \frac{c_{m}^{2}}{g} \\ h_{ds} \end{array}\right. },\quad u(x,t) = {\left\{ \begin{array}{ll} 0\quad m/s &{}\text {if } x \le x_{A}(t) \\ \frac{2}{3}(\frac{x}{t}+\sqrt{g\,h_{up}}) &{} \text {if } x_{A}(t)\le x \le x_{B} (t) \\ 2(\sqrt{g\,h_{up}}-c_{m}) &{} \text {if } x_{B}(t)\le x \le x_{C} (t) \\ 0\quad m/s &{}\text {if } x_{C}(t) \le x, \end{array}\right. } \end{aligned}$$

where *x* denotes the axial position, $x_{A}(t)=x_{0}-t\sqrt{g\,h_{up}}$, $x_{B}(t)=x_{0}+t(\sqrt{g\,h_{up}}-3c_{m})$ and $x_{C}=x_{0}+t\frac{2c_{m}^{2}(\sqrt{g\,h_{up}}-c_{m})}{c_{m}^{2}-g\,h_{ds}}$, and where $c_{m}=\sqrt{gh_{m}}$ denotes the selected solution of $-8gh_{ds}c_{m}^{2}(g\,h_{up}-c_{m}^{2})^{2}+(c_{m}^{2}+g\,h_{ds})(c_{m}^{2}-g\,h_{ds})^{2}=0$.

The upstream water level ($h_{up}$) is considered as an input random variable whose values are uniformly sampled within its plausible variability range $h_{up}\in \mathcal {U}\left[ 8,\;11\right]$, whereas the downstream water depth is kept constant at a deterministic value $h_{ds}=1\,m$. For each selected value in the generated sample set of the upstream water level, the analytical solution, given by Eq. (14), is evaluated over the $N_{x}=1\,000$ nodes that contain the computational domain $x\in \left[ 0,\;100\right] m$ for all the $N_{t}=104$ time-steps of the temporal domain $t\in \left[ 1,\;4\right] s$. The obtained solution vectors are then concatenated to construct the so-called high-fidelity snapshot matrix that trains the proposed non-intrusive reduced-order model (NIROM-CAEs) during the offline training phase.

**Fig. 8:**
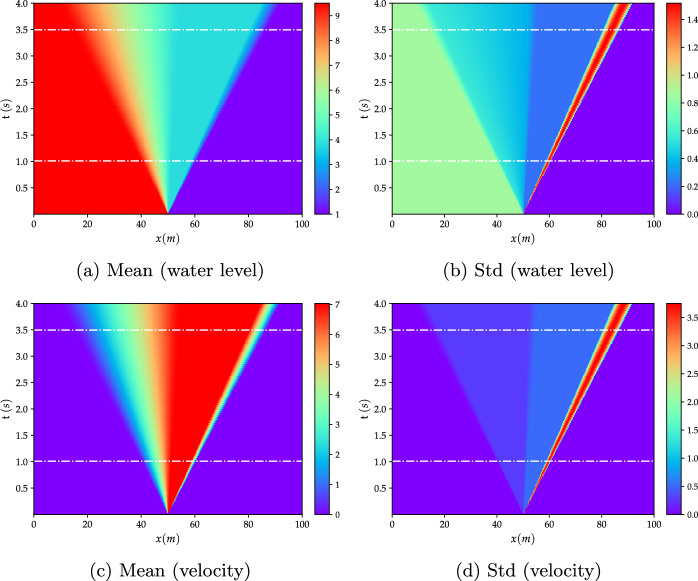
Contour plots of the mean (left column) and standard (right column) deviation of the Stoker’s analytical solution of the water level (upper row) and the velocity (bottom row) obtained from the LHS solution with 5000 realizations. The two horizontal dashed lines represent the times for which the results in Figs. 9 and 10 are presented

Stoker’s problem is considered among the most challenging benchmark test cases due to its strong hyperbolic behavior and the discontinuity accompanying the propagation of the front wave resulting from the initial breaking. The performance of the proposed NIROM-CAEs is assessed over this test case by comparing the obtained statistical moments to those from the LHS reference solution, whose contours for the water level and velocity fields are depicted in the spatiotemporal plane as shown in Fig. 8, where the longitudinal and transversal axes represent the spatial and temporal dimensions, respectively. The two horizontal lines show the time locations, $t\approx 1$ and $t\approx 3.5$ s, for which the evolution of the mean and standard deviation profiles over the channel length, respectively, are compared with the LHS and POD-ANN results.

Similar to the former numerical test case, the spatial and temporal autoencoders structures are composed of three 1D-convolutional layers with 32, 64 and 128 channels each, thereby reducing the spatial dimension from $N_{x}=1\,000$ to $L_{x}=50$, and then to $L_{t}=10$, representing the reduced latent space. The obtained spatiotemporal latent space is then linked with the input parameter vector via MLP mapping. The detailed architectures of the proposed NIROM-CAEs model with its CAE-space, CAE-time, and MLP are summarized in Tables 2, 3 and 6, respectively. The convergence history of the spatial, temporal CAEs and the MLP during the training phase are depicted in Fig. 19 with the number of epochs of 500, 1000, and 3000, respectively.

**Fig. 9:**
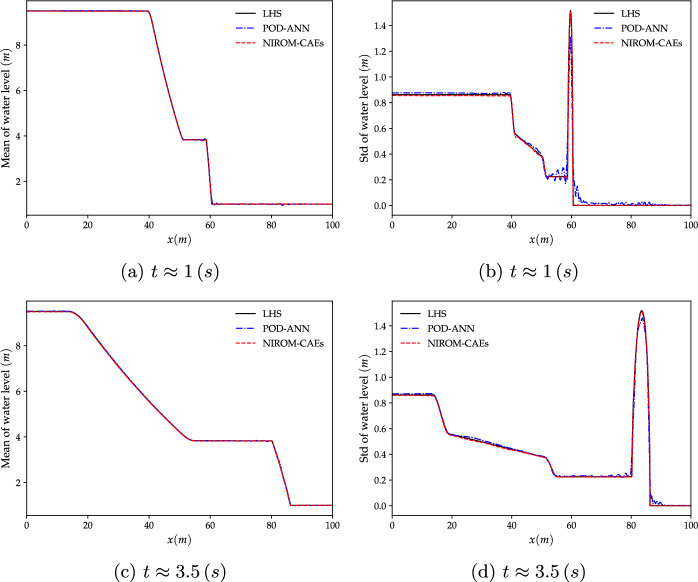
Distribution of the mean (left) and standard deviation (right) of the water level along the channel length at different time steps ($t\approx 1$ and $t\approx 3.5\; s$). The results obtained from the POD-ANN and NIROM-CAEs techniques are compared with those from the LHS reference solution

**Fig. 10:**
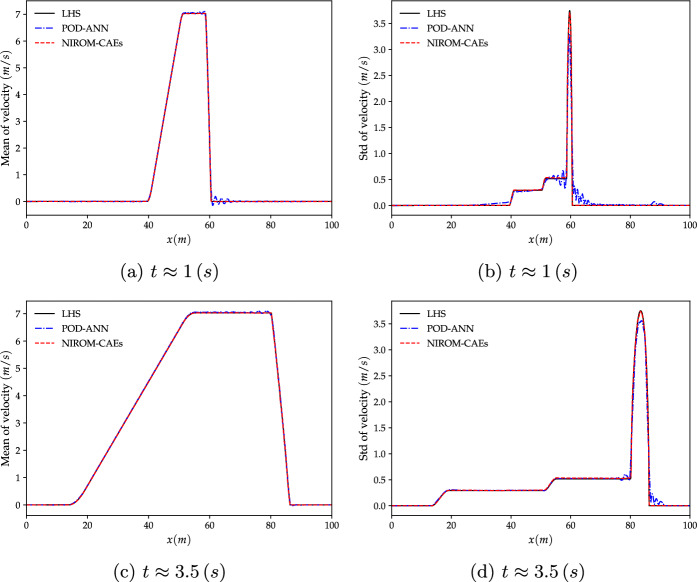
Distribution of the mean (left) and standard deviation (right) of the velocity along the channel length at different time steps ($t\approx 1$ and $t\approx 3.5\; s$). The results obtained from the POD-ANN and NIROM-CAEs techniques are compared with those from the LHS reference solution

The results obtained by the proposed NIROM-CAEs model (presented in terms of the variation of the mean and standard deviation profiles as a function of the *x*-coordinate at different times ($t\approx 1,\, 3.5\;s$) are compared with those from the LHS reference solution (obtained with $5\,000$ realizations), as shown in Figs. 9 and 10. In addition to the LHS solution, the POD-ANN model is considered another solution with which to assess the predictive ability of the nonlinear NIROM-CAEs. The structure of the neural network introduced in the POD-ANN is constituted by three hidden layers, each of which contains 50 neurons. A sample set of $N_{s}=300$ values of the upstream water level is randomly generated in its plausible variability range to build the snapshot matrices for both models by collecting the corresponding high-fidelity solutions, of which $80\%$ is used for training and the remaining $20\%$ for validation.

The comparison of the mean and standard deviation profiles of the water level presented in Fig. 9 shows that both the POD-ANN and NIROM-CAEs models accurately predict the distribution of the mean over the whole channel for the two simulation times, as a good agreement with the reference LHS profiles can be observed. However, the POD-ANN predictions for the standard deviation exhibit spurious oscillations in the area surrounding the front shock wave, where significant deviations appear in comparison with the profiles from the LHS solution. In contrast, the predicted profiles from the proposed NIROM-CAEs show an excellent agreement with those from the high fidelity LHS solutions, even in the area where the discontinuity occurs. The same trend can be observed in the velocity statistical moments’ profiles, as shown in Fig. 10, conversely to the approximated outputs from the POD-ANN, which are characterized by an oscillatory behavior. It can be concluded that, for this test case, the use of convolutional neural network autoencoders with nonlinear activation functions provides a powerful nonlinear compression model with high predictive abilities in capturing the dynamics of the outputs in contrast with the linear compression model based on the traditional POD approach.

**Fig. 11:**
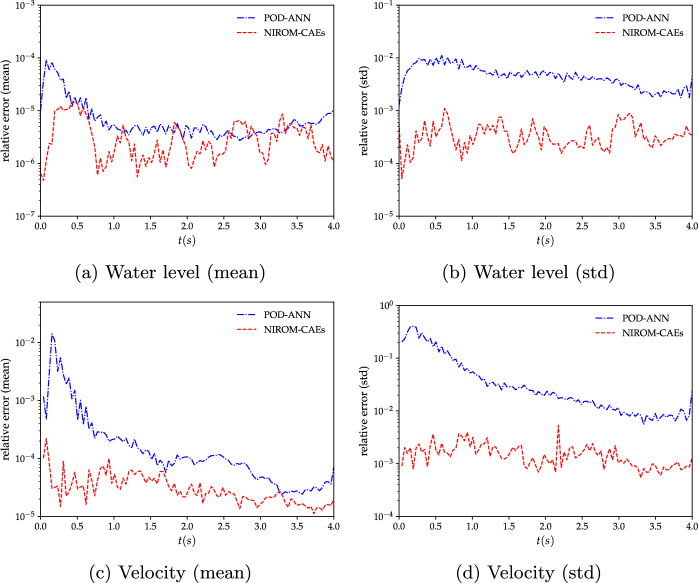
Comparison of the relative $L^{2}$-error profiles of the mean (left) and standard deviation (right) of the water level (first row) and velocity (second row) as a function of time, obtained with the POD-ANN and NIROM-CAEs. Errors are evaluated with respect to the 5000-realization LHS reference solution

To better illustrate the potential of nonlinear model reduction approaches to efficiently predict the statistical moments of the output responses of high-complexity problems, an evaluation of the relative $L^{2}$ error is performed for both the water level and velocity fields, as shown in Fig. 11. The mean and the standard deviation errors are calculated for each time step by evaluating the differences between the predicted statistical from the LHS reference over the entire calculation domain, as formulated in Eq. (13). The evolution of the relative errors as a function of time shows that the NIROM-CAEs approach presents a better approximation of the output responses, with errors on the order of $10^{-7}$ for the mean and $10^{-4}$ for the standard deviation, unlike the linear POD-ANN technique which presents higher error values that can reach up to two orders of magnitude in the case of the standard deviation for both velocity and water level. This quantitative analysis shows that the introduction of non-linearities contributes to a better approximation of the statistical moments, allowing the construction of efficient spatiotemporal compression models for time-dependent complex problems.

### Application to a hypothetical dam-break in a river

**Fig. 12:**
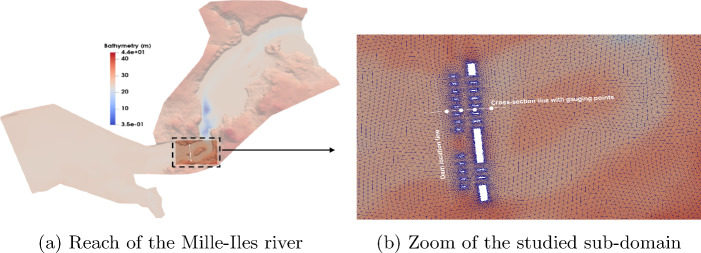
Sketch of the reach of the Mille Iles river with a close-up view of the studied zone. The cross-section line and the gauging points indicate locations where results are represented as a function of the longitudinal direction and time, respectively

The proposed approach is applied to a third test case to evaluate its ability to perform an efficient uncertainty propagation analysis on a spatial domain with complex bathymetry. The test concerns a reach of the Milles-Iles River (province of Québec, Canada) including a dam as shown in Fig. 12. The data relating to the bathymetry and the roughness coefficient were provided by the Communeauté Métropolitaine de Montréal (CMM) from measurements and observations. The sub-domain of study is composed of an unstructured triangular mesh with $16\,763$ elements and $N_{e}=10\,200$ nodes, over which high-fidelity solutions of the quantities of interest are collected from an in-house multi-GPU finite volume solver for shallow water equations [45]. A detailed description of the physical domain of this test case was addressed in [13].

A fictitious breaching process was initialized on both parts of a hypothetical dam with unequal water levels, located as indicated by a line in Fig. 12b. The downstream part of the dam is considered as dry whereas the free surface of the upstream part is considered as a random input parameter whose values are uniformly generated within its plausible variability range $\eta _{up}\in \mathcal {U}\left[ 29,\;32\right] \,m$. The snapshot matrix is obtained by running the numerical solver for each value of the upstream free surface, selected randomly from the generated sample set, for the whole $N_{t}=100$ simulation time steps that constitute the temporal domain ($t\in \left[ 0,\, 50\right] \,s$). For each parameter-time combination, a so-called high-fidelity solution is stored in a vector of dimension $N_{x}=10\,200$, representing the free surface values at each node of the computational domain. The concatenation of these solution vectors thus allows the construction of the snapshot matrix.

The spatial compression is performed by the proposed NIROM-CAEs using a space-autoencoder composed of two 1D-convolutional layers, one with 32 and the other one with 64 channels, followed by a max-pooling and dense layers, thereby reducing the initial spatial dimension from $N_{x}=10\,200$ nodes to $L_{x}=50$, representing the latent dimension. A second time-autoencoder is then applied to the generated spatial latent space to reduce the temporal dimension from $N_{t}=100$, representing the number of time-steps, to $L_{t}=10$. The time-autoencoder has a succession of three 1D-convolutional max-pooling layers followed by a dense layer that links the channels to the temporal latent space. The obtained latent space is then linked to the input parameter through an MLP of three layers, each with a width of 128 neurons, to map the values of the input parameter to the final latent space. A detailed description of the architectures of the space and time autoencoders and the MLP are presented in Tables 4, 5, and 6. The numbers of trainable parameters are $6\,915\,197$, $153\,230$ and $34\,570$, respectively. The convergence histories of the space and time CAEs and the MLP during the training phase, performed on a Tesla P100 GPU with 32 GB of memory, are depicted in Fig. 20 with the number of epochs of 200, 1000 and 2000, respectively.

**Fig. 13:**
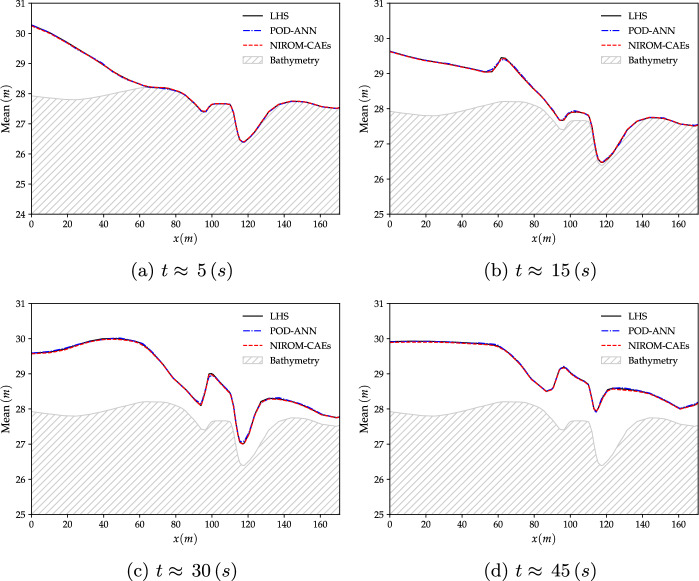
The mean profiles of the water level over the cross-section line at various time-steps, obtained with the POD-ANN and NIROM-CAEs and compared with those from the LHS reference solution (with 2000 realizations). **a**: $t\approx 5\,s$, **b**: $t\approx 15\,s$, **c**: $t\approx 30\,s$ and (**d**): $t\approx 45\,s$

**Fig. 14:**
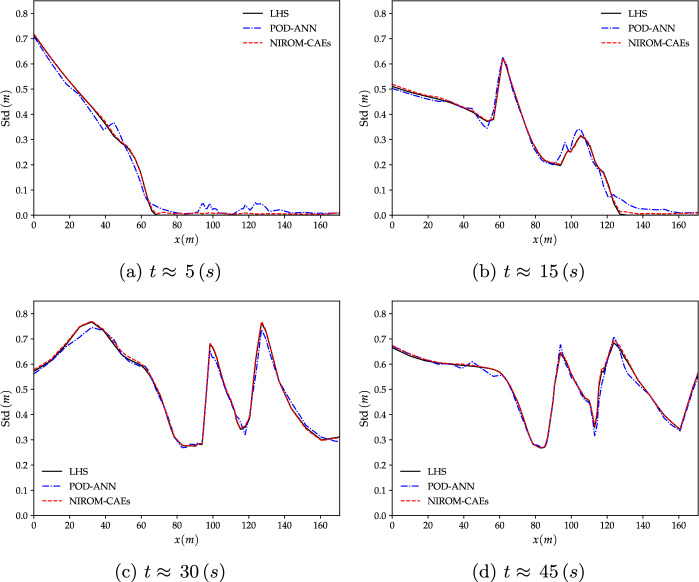
Comparison of the standard deviation profiles of the water level over the cross-section line at various time-steps; the POD-ANN and NIROM-CAEs profiles vs those from the LHS reference solution (with 2000 realizations). **a**: $t\approx 5\,s$, **b**: $t\approx 15\,s$, **c**: $t\approx 30\,s$ and (**d**): $t\approx 45\,s$

**Fig. 15:**
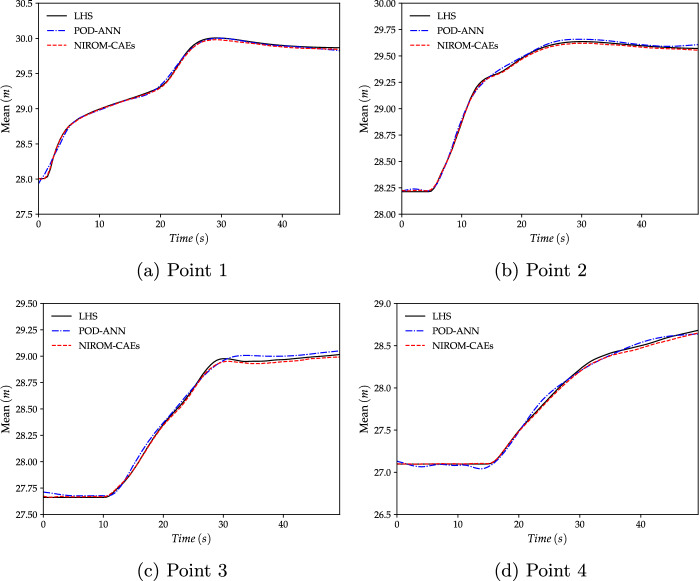
Evolution of the mean profiles of the water level as a function of time at four gauging points. The profiles obtained with the NIROM-CAEs are compared to those from the POD-ANN technique and the LHS reference solution (with $2\,000$ realizations). **a**: Point 1, **b**: Point 2, **c**: Point 3 and **d**: Point 4

**Fig. 16:**
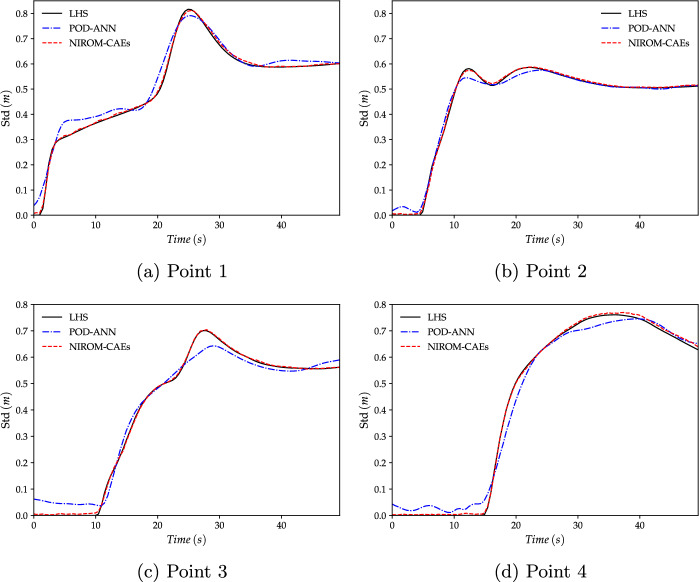
Evolution of the standard deviation profiles of the water level as a function of time at four gauging points. The profiles obtained with the NIROM-CAEs are compared to those from the POD-ANN technique and the LHS reference solution (with 2000 realizations). **a**: Point 1, **b**: Point 2, **c**: Point 3 and **d**: Point 4

The results stemming from this test case are presented mainly in terms of the variation of the mean and standard deviation profiles of the water surface level over the studied computational sub-domain. The results obtained with the proposed NIROM-CAEs are compared with those from the LHS solutions, both obtained by running the deterministic numerical solver using a sample set of $N_{s}=2000$ points of the uncertain initial upstream water level. The predictive model of the POD-ANN approach is based on a neural network composed of three hidden layers, each with of 50 neurons, that map the reduced-basis coefficient to the input parameter. Figures 13 and 14 present the variation of the mean and the standard deviation profiles, respectively, as a function of the longitudinal coordinate along the cross-section line, as shown in Fig. 12b. The comparison of the predicted mean profile of the water level at different simulation times shows that the NIROM-CAEs’predictions are in good agreement with the profiles from the LHS reference solutions. The POD-ANN model also presents satisfactory predictions for the mean. The shock wave propagation is visible as the water level over the cross-section line increases with time. Figure 13a through 13d show the hatched area that represents the bathymetry of the terrain. Despite the accurate results obtained by the POD-ANN approach for the mean profiles of the water level, its standard deviation predictions over the cross-section line reveal spurious oscillations, as shown in Fig. 14a through 14d. In contrast, the predictions from the NIROM-CAE approach show good agreement with the reference solution for all the presented simulation times. This further illustrates the good abilities of the proposed nonlinear reduced-order approach in approximating the outputs of high-dimensional time-dependent problems.

In addition to the results presented above, four gauging points were chosen to depict the evolution of statistical moments as a function of time. These gauging points, whose approximate positions on the cross-section line are shown in Fig. 12b, were selected to show the ability of the proposed nonlinear technique to capture the temporal dynamics. In Figs. 15 and 16, the time variations of the mean and standard deviation are presented at the four selected gauging points. The results from NIROM-CAEs, POD-ANN, and the LHS solution can be compared. These plots show the increase in the water level from point 1 (close to the dam location) to point 4 (far from the dam), which allows an estimation of the arrival time of the flooding wave. Slight deviations can be observed in the mean profiles from the POD-ANN at almost all the gauging points, while the profiles from NIROM-CAEs present a good match with the reference solution, as shown in Fig. 15a through 15d. This tendency is further confirmed by the standard deviation profiles, where excellent superimposition can be observed between the profiles from the NIROM-CAEs and those from the LHS solution at all four gauging points. Conversely, the predictions from the linear POD-ANN technique present remarkable deviations with poor accuracy in the approximations of the standard deviation profiles of the water level at all gauging points, as indicated in Fig. 16a through 16d.

**Fig. 17:**
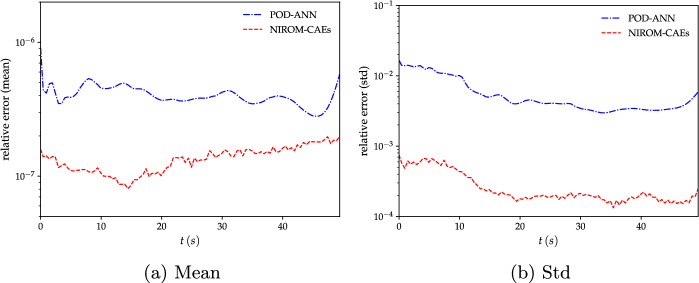
Comparison of the relative $L^{2}$-error of the mean and standard deviation of the water level as a function of time based on the POD-ANN technique and the proposed NIROM-CAEs approach. Errors are computed with respect to the reference LHS solution (with $N_{s}=2000$ realizations)

**Fig. 18:**
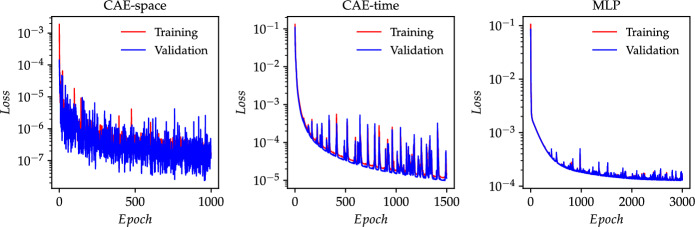
Evolution of the training and validation losses of CAEs and MLP for the Burgers equation test case

**Fig. 19:**
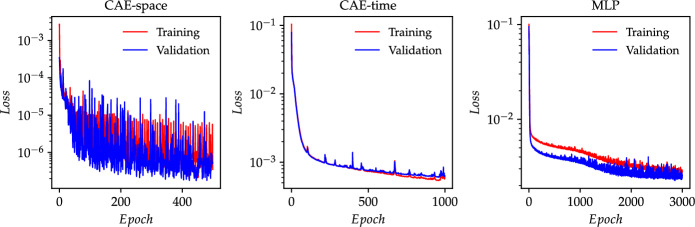
Evolution of the training and validation losses of CAEs and MLP for the Stoker’s analytical solution test case

**Fig. 20:**
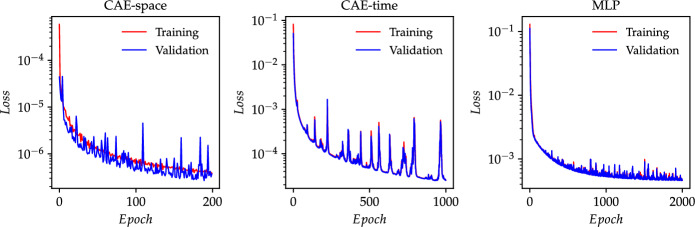
Evolution of the training and validation losses of CAEs and MLP for the hypothetical dam break of the Mille-Iles river

For a better insight into the accuracy of both reduced-order model approaches, namely POD-ANN and NIROM-CAEs, a quantitative evaluation is performed by computing the relative $L^{2}$-error over the whole computational domain (with the LHS reference sampling solution obtained with $N_{s}=2000$ points). Thus, the relative error of the mean and standard deviation is represented as a function of time, as shown in Fig. 17. These plots show that the proposed NIROM-CAEs have much lower values of the relative error for both the mean and the standard deviation. The effect of the training set sample size ($N_{s}$) on the maximum relative error over time is reported in Table 1. Three values of the sample size $N_{s}=30,\,90$ and 300 were tested, and for each value, the maximum $L^{2}$-error was computed over time for both the mean and the standard deviation. It is clear that as the sample size increases, the relative error of the NIROM-CAEs decreases faster than that of the POD-ANN, particularly for the standard deviation, where a difference in the error values can reach almost two orders of magnitude. This quantitative analysis supports the above-mentioned observations concerning the predictive abilities of the proposed NIROM-CAEs. Another meaningful result from Table 1 is the dimension of the generated latent space, which is considerably reduced for the NIROM-CAEs, on the order of $L_{x}=50$ and $L_{t}=10$, independently of the sample size, conversely to the POD-ANN, whose dimension of the reduced basis increases from $L_{POD}=800$ for $N_{s}=30$ to reach $L_{POD}=3337$ for $N_{s}=300$, which may present a challenging task for the available computational capacities.

## Conclusion

This paper presents a non-intrusive reduced-order model based on convolutional autoencoders (NIROM-CAEs) for parameter-varying and time-dependent fluid problems. The model is fully data-driven and exploits the nonlinear framework provided by the convolutional autoencoders to tackle physical problems presenting a high degree of complexity. The construction of the offline training stage of the proposed approach consists of two successive compression levels along the spatial and temporal dimensions. The first encoder (a space encoder) performs compression along the spatial dimension of the input snapshot matrix to generate the encoded low-dimensional latent space. A second encoder (a time encoder), whose structure is similar to that of the former, applies convolutional operations along the temporal dimension of the encoded space to generate a second spatiotemporal latent vector with much lower dimensions. The encoded latent space thus obtained is used as the output data for a multilayer perceptron (MLP), deployed at the bottom level to train the mapping with the inputs from the parametric space. Once the offline training stage is constructed, the online predictive stage consists of the generation of a new set of unseen points in the design space to provide the trained MLP at the bottom level. The resulting spatiotemporal latent vectors are then decoded through successive 1D decoders to predict solutions describing the high-dimensional spatiotemporal dynamics of the output quantities of interest. The proposed reduced-order model allows for rapid and accurate predictions of the statistical moments of the output distributions, thus enabling uncertainty propagation analyses.

The performance and accuracy of the proposed reduced-order model (NIROM-CAEs) are demonstrated on three nonlinear examples parametrized by a random variable with an appropriate variability range. The two first test cases concern the Burgers and Stoker’s solutions, known as challenging test cases presenting a high hyperbolic behavior or a discontinuity accompanying the shock wave. The numerical results show that the model offers accurate approximations of the statistical moments in comparison to those of the reference solutions from the Latin Hypercube Sampling approach (LHS) method, unlike the linear POD-ANN model which shows some limitations in reproducing the dynamics of the outputs where an oscillatory behavior is observed in the predicted profiles. The results also reveal the low relative error of the proposed NIROM-CAEs, further highlighting its abilities. The model is then applied to a third case to analyze uncertainty propagation in a dam break flow over a real terrain. The NIROM-CAEs proved that its accuracy in the reconstruction of the mean and the standard deviation profiles, where good concordances with those from the LHS reference solutions were observed. The predictions from the POD-ANN show spurious oscillations, particularly for the temporal evolution at gauging points. Thus, the proposed non-intrusive reduced-order model based on convolutional autoencoders presents a powerful tool for nonlinear dimensionality reduction for parameter-varying, time-dependent, and large-scale problems characterized by strong hyperbolic behavior or even with the presence of a discontinuity. The model offers the construction of accurate surrogate predictions at a low cost.

**Table 1:** Effect of the sample size $N_{s}$ on the maximum relative error in the $L^{2}$-norm for the mean and standard deviation obtained from the NIROM-CAEs ($L_{x}=50$, $L_{t}=10$) and POD-ANN ($\epsilon _{s}=\epsilon _{t}=10^{-8}$) approaches. Errors are computed with respect to the LHS reference solution (with $N_{s}=2000$ realizations)

$N_{s}$	$L_{POD}$	$Err_{L^{2},\,Mean}^{max}$		$Err_{L^{2},\,Std}^{max}$	
		POD-ANN	NIROM-CAEs	POD-ANN	NIROM-CAEs
30	860	8.2855E−07	2.1896E−06	0.024537	0.010349
90	2112	6.0781E−07	9.0018E−07	0.013303	0.005838
300	3387	9.1037E−07	1.9986E−07	0.016687	7.4981E−04

## Data Availability

The software can be shared upon request.

## A Training convergence history and models’ configurations

See Tables 2, 3, 4, 5, 6 and Figs. 18, 19, 20.

**Table 2:** CAE-space architecture for Burger’s and Stoker’s test cases

**Layer**	**Nb of filters**	**Kernel size**	**Activation function**	**Output shape**
*Encoder-space*				
Input	–	–	–	$1000\times 1$
Conv-pool	32	3–2	PReLU	$500\times 32$
Conv-pool	64	3–2	PReLU	$250\times 64$
Conv-pool	128	3–5	PReLU	$50\times 128$
Flatten	–	–	–	6400
Dense	–	–	PReLU	60
Dense (output)	-	-	PReLU	$L_{x}=50$
*Decoder-space*				
Input	–	–	–	$L_{x}=50$
Dense	–	–	PReLU	60
Dense	–	–	PReLU	6400
Reshape	–	–	–	$50\times 128$
Conv-Upsamp	128	3–5	PReLU	$250\times 128$
Conv-Upsamp	64	3–2	PReLU	$500\times 64$
Conv-Upsamp	32	3–2	PReLU	$1000\times 32$
Conv (output)	1	3	PReLU	$1000\times 1$

**Table 3:** CAE-time architecture for Burger’s and Stoker’s test cases

**Layer**	**Nb of filters**	**Kernel size**	**Activation function**	**Output shape**
*Encoder-time*				
Input	–	–	–	$104\times L_{x}$
Conv-pool	32	3–2	PReLU	$52\times 32$
Conv-pool	64	3–2	PReLU	$26\times 64$
Conv-pool	128	3–2	PReLU	$13\times 128$
Flatten	–	–	–	$1\,664$
Dense (output)	–	–	PReLU	$L_{t}=10$
*Decoder-time*				
Input	–	–	–	$L_{t}=10$
Dense	–	–	PReLU	1664
Reshape	–	–	–	$13\times 128$
Conv-Upsamp	128	3–2	PReLU	$26\times 128$
Conv-Upsamp	64	3–2	PReLU	$52\times 64$
Conv-Upsamp	32	3–2	PReLU	$104\times 32$
Conv (output)	1	3	PReLU	$104\times L_{x}$

**Table 4:** CAE-space architecture for hypothetical dam break of Miles-Iles river

**Layer**	**Nb of filters**	**Kernel size**	**Activation function**	**Output shape**
*Encoder-space*				
Input	–	–	–	$10,200\times 1$
Conv-pool	32	3–5	PReLU	$2040\times 32$
Conv-pool	64	3–5	PReLU	$408\times 64$
Flatten	–	–	–	26112
Dense	-	–	PReLU	120
Dense (output)	–	–	PReLU	$L_{x}=50$
*Decoder-space*				
Input	–	–	–	$L_{x}=50$
Dense	–	–	PReLU	120
Dense	–	–	PReLU	$26\,112$
Reshape	–	–	–	$408\times 64$
Conv-Upsamp	64	3–5	PReLU	$2040\times 64$
Conv-Upsamp	32	3–5	PReLU	$10200\times 32$
Conv (output)	1	3	PReLU	$10200\times 1$

**Table 5:** CAE-time architecture for hypothetical dam break of Miles-Iles river

**Layer**	**Nb of filters**	**Kernel size**	**Activation function**	**Output shape**
*Encoder-time*				
Input	–	–	–	$100\times L_{x}$
Conv-pool	32	3–2	PReLU	$50\times 32$
Conv-pool	64	3–2	PReLU	$25\times 64$
Conv-pool	128	3–5	PReLU	$5\times 128$
Flatten	–	–	–	640
Dense (output)	–	–	PReLU	$L_{t}=10$
*Decoder-time*				
Input	–	–	–	$L_{t}=10$
Dense	–	–	PReLU	640
Reshape	–	–	–	$5\times 128$
Conv-Upsamp	128	3–5	PReLU	$25\times 128$
Conv-Upsamp	64	3–2	PReLU	$50\times 64$
Conv-Upsamp	32	3–2	PReLU	$100\times 32$
Conv (output)	1	3	PReLU	$100\times L_{x}$

**Table 6:** MLP architecture for Burgers, Stoker’s test cases and hypothetical dam break

**Layer**	**Activation function**	**Output shape**
*MLP*		
Input	ReLU	1
Dense	ReLU	128
Dense	ReLU	128
Dense	ReLU	128
Dense (output)	Linear	$L_{t}=10$
